# Efficient Triazine Derivatives for Collagenous Materials Stabilization

**DOI:** 10.3390/ma14113069

**Published:** 2021-06-04

**Authors:** Vanessa Gatto, Silvia Conca, Noemi Bardella, Valentina Beghetto

**Affiliations:** 1 Department of Molecular Sciences and Nanosystems, University Ca’ Foscari of Venice, Via Torino 155, 30172 Mestre, VE, Italy; vanessa.gatto@unive.it (V.G.); silvia.conca@unive.it (S.C.); noemi.bardella@unive.it (N.B.); 2 Crossing S.r.l., Viale della Repubblica 193/b, 31100 Treviso, TV, Italy

**Keywords:** triazine amidation agents, collagen cross-linking, green chemistry, sustainable leather tanning

## Abstract

Nowadays, the need to reduce plastic waste and scantly biodegradable fossil-based products is of great importance. The use of leather as an alternative to synthetic materials is gaining renewed interest, but it is fundamental that any alternative to plastic-based materials should not generate an additional environmental burden. In the present work, a simple protocol for collagen stabilization mediated by 2-chloro-4,6-diethoxy-1,3,5-triazine (CDET) and a *tert*-amine has been described. Different *tert*-amines were tested in combination with CDET in a standard amidation reaction between 2-phenylethylamine and benzoic acid. Best performing condensation systems have been further tested for the cross-linking of both collagen powder and calf hides. The best results were achieved with CDET/NMM giving high-quality leather with improved environmental performances.

## 1. Introduction

Since 1907 when Leo Baekeland invented Bakelite, the first fully synthetic plastic, over 8300 Mt of fossil-based polymers have been produced, 4900 Mt of which were landfilled, incinerated, or dispersed in the environment. The wide use of plastic materials derives from their technical characteristics, high versatility, lightweight, and low price. Most of the plastic materials commercialized today are still produced from fossil sources [1] and fossil-based plastics are scantly biodegradable, so that the environment and humans are increasingly exposed to contamination deriving from plastic waste [2,3,4,5,6]. At the same time, the demand for fossil-based products is constantly increasing, not only for plastic production, but also for many other applications. In this scenario, bio-based polymers are becoming an increasingly attractive and eco-friendly alternative to fossil-based polymers [3,7,8,9,10,11]. The global market’s growing trend for green polymers and bioplastic packaging materials is expected to reach $51.2 billion by 2023 [12].

Hides and leather processing is one of mankind’s oldest processes, allowing us to recover waste from the food industry to produce garments and other products. Since the advent of plastics, leather goods have been increasingly replaced by synthetic polymers such as polyurethane and polyvinylchloride-based materials. As synthetic polymers were gaining growing interest in the 1970–1980s, the possibility to substitute polluting processes with fossil-based materials appeared a sustainable and winning solution. For this reason, synthetic leather is often referred to as eco-leather, suggesting a lower environmental impact compared to leather from hides. The global synthetic leather market size was valued at USD 29.3 billion in 2019 and is projected to expand at an annual rate of 4.4% from 2020 to 2027 [13]. Globally, increasing demand from the footwear sector is expected to play a key role in boosting the overall market growth. 

Leather processing has a high environmental impact since many steps are required to transform raw hides into finished good quality leather. Over 2 kg of chemicals (30% hazardous, DIR 67/548 CEE) and 11–15 m^3^ of water are required to produce one square meter of finished leather, generating 3 kg of solid/liquid waste (400,000 t of solid waste yearly in the E.U.) [14]. To date, over 85% of leather processed worldwide is chrome tanned. This predominant use of Cr (III) salts for hides processing derives from the high-quality of leather produced and moderate price.

When considering the present need to reduce plastic waste and scantly biodegradable fossil-based products, the use of leather as an alternative to synthetic leather and other plastic products is gaining renewed interest. It is, nevertheless, fundamental that any alternative to plastic-based materials should not generate an additional environmental burden, shifting the problem from plastic waste to chrome-containing slurries. The implementation of environmentally sustainable manufacturing processes may act as a strong promoter for the use of collagen as a viable alternative to plastic-based materials.

High chromium exhaustion tanning technologies have been studied to reduce the concentration of heavy metals in slurries and wastewater; nevertheless, they do not eliminate heavy metals [15,16,17,18,19,20,21,22,23,24,25,26]. On the other hand, metal-free tanning agents employed to produce “wet-white” leather (for example, aldehydes and tannins) often have limited applications compared to chrome tanned leather (“wet-blue” leather) since their physical–mechanical characteristics are generally lower compared to wet-blue leather [27,28].

Triazine tanning agents are gaining increasing interest in recent years as valid, environmentally friendly alternatives that are metal-, formaldehyde-, and phenol-free [15,29,30,31,32]. Presently the leather industry is suffering from restrictions due to the ECHA-REACH regulation, which has significantly limited the variety of chemical substances allowed as tanning agents. Formaldehyde has been restricted since January 2016 for its assessed toxicity and carcinogen activity and is no longer used as a tanning agent, while it is still used for the synthesis of phenol-formaldehyde-based synthetic tannins. Glutaraldehyde has substituted formaldehyde as a tanning agent; nevertheless, very recently (January 2021), its use has also been restricted as carcinogenic together with 4,4’-sulfonyldiphenol, one of the most common constituents of synthetic tannins as an endocrine disruptor.

Due to the REACH restrictions, in a relatively short time, very few alternatives to chrome salts will be left on the market, with all the environmental and socio-economic impacts that chrome salts generate. Thus, there is a real need for more sustainable practices, and triazine tanning agents appear particularly attractive for their low cost, reduced environmental impact, and high-quality of the leather produced.

Tanning agents react with the collagen of animal hides by generating cross-linking bonds, improving its chemical and physical properties, and transforming a biodegradable material into a stable product. The nature and strength of the chemical bonds formed are different depending on the reactant used and the collagen functional groups involved, so different tanning agents convey different characteristics to the final product [28]. Shrinkage temperature (Ts) may be used to establish the effectiveness of a tanning agent since Ts is correlated to the cross-linking density within the hide and its stability [33,34].

For example, tanning with 5 %wt basic chromium sulphate by the weight of the hide processed gives leather with Ts among the highest known today (≥100 °C), while other tanning agents give Ts values around 75–78 °C even if used in 20–25 %wt by wt of the hide processed [28,33].

Our research group has long been involved in the study of innovative, sustainable processes for the industry and leather processing [29,35,36]. In the last years, 4-(4,6-dimethoxy-1,3,5-triazin-2-yl)-4-methyl-morpholinium chloride (DMTMM) has found many applications as dehydro-condensation agent for collagen cross-linking [29,30,37], amine grafting on hyaluronan [38], amide, and peptide synthesis [39,40,41,42,43]. DMTMM is a very efficient cross-linking agent which promotes the reaction between carboxylic and amine groups in mild reaction conditions to form amides.

Nevertheless, DMTMM has moderate stability in time, is scantly available, and expensive for the specific application. With all of this in mind, we believe that the disadvantages encountered using triazine quaternary ammonium salts such as DMTMM could be overcome by the direct addition of a 2-halo-4,6-dialkoxy-1,3,5-triazine and a *tert*-amine in the reaction mixture or the tanning bath. This straightforward methodology avoids the synthesis of isolated DMT-Ams(X), reduces production complexity, solvent use, and allows us to achieve a library of tanning agents modulated according to specific requirements.

Few examples of the use of 2-chloro-4,6-dimethoxy-1,3,5-triazine (CDMT) in the presence of *tert*-amines have been previously reported as condensation agents, and to the best of our knowledge, no other 2-chloro-4,6-dialkoxy-1,3,5-triazine has ever been tested as a condensation or tanning agent [31,44].

**Figure 1:**
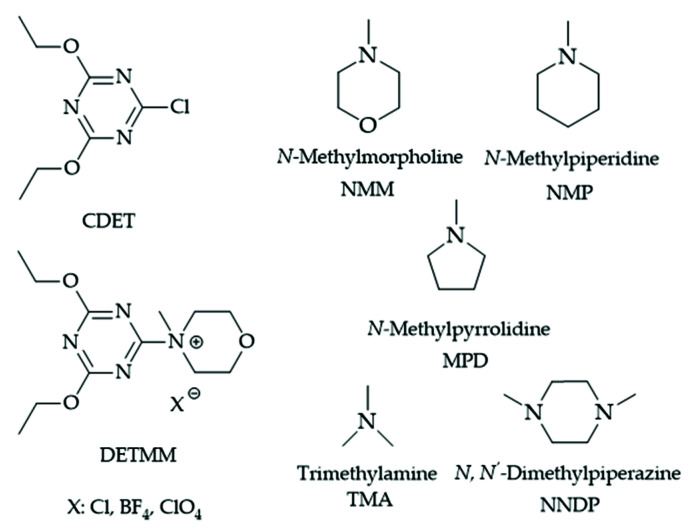
Chemical structure of CDET, DETMM(X), and different amine tested.

Thus, in this work, 2-chloro-4,6-diethoxy-1,3,5-triazine (CDET) and different *tert*-amines (see Figure 1) have been tested first as condensation agents for the synthesis of amides, and then, best-performing CDET/*tert*-amine employed as tanning agents of different collagen matrixes (collagen powder and calf hides). Environmental benefits, such as reduced chemical hazard and chemical consumption, compared to conventional tanning systems, have also been evaluated.

## 2. Materials and Methods

### 2.1. Materials

We synthesized 2-chloro-4,6-diethoxy-1,3,5-triazine (CDET) as reported in literature [45]. Benzoic acid, *N*-phenethylbenzamide, *tert*-amines, mesitylene, solvents, and all other chemicals were purchased from Sigma Aldrich (St. Louis, MO, USA) and used without any purification. Bovine collagen powder was purchased from Filk (Research Institute of Leather and Plastic Sheeting, Freiberg, Germany). Delimed calf hides were obtained from tanneries of the Santa Croce sull’Arno Leather District.

### 2.2. Methods

GLC analyzes were performed using an Agilent Technologies 6850 gas chromatograph equipped with an FID detector and Agilent HP-1 capillary column (30 m × 0.32 mm × 0.25 µm). Analysis conditions: T 50 °C for 1 min., rate: 20 °C/min., T 230 °C for 15 min. ^1^H-NMR spectra were recorded on a Bruker Avance AC 300 spectrometer (Billerica, MA, USA) operating at 300.21 and 75.44 MHz, respectively. The chemical shift values of the spectra are reported in δ units with reference to the residual solvent signal. IR spectra were collected in the range 4000–400 cm^−1^ using a PerkinElmer Spectrum One spectrophotometer. Differential scanning calorimetry (DSC) of collagen samples was performed on a Netzsch STA 409 cell (Selb, Germany) fitted with an air-cooling compressor at ambient temperature and a controller Netzsch TASC 414/3. The instrument temperature was calibrated using indium as the standard. Collagen samples (about 7.0 mg) were weighed into an aluminum oxidized melting pot and sealed. Samples were heated from 30 °C to 120 °C at a scanning rate of 10 °C/min. A sealed melting pot filled with Al_2_O_3_ (about 7.0 mg) was used as the reference.

#### 2.2.1. General Procedure for Dehydro-Condensation Reactions with CDET/*tert*-Amine System

Benzoic acid (158.7 mg, 1.3 mmol) was dissolved in solvent (methanol, 6 mL) and subsequently CDET (264.7 mg, 1.3 mmol), *N*-methylmorpholine (NMM) (131.5 mg, 1.3 mmol) and 2-phenylethylamine (145.4 mg, 1.2 mmol) was added. After 15 min and 1 h, yield in amide *N*-phenethylbenzamide was monitored by GLC using mesitylene as internal standard (1.2 mmol). The reaction mixture was filtered and the yellowish solid recovered was dissolved in CH_2_Cl_2_ and extracted with water (3 × 30 mL) to isolate *N*-phenethylbenzamide. The combined organic phases were dried over MgSO_4_ filtered and concentrated in vacuo to give III as a white solid.

#### 2.2.2. Synthesis of 4-(4,6-diethoxy-1,3,5-triazin-2-yl)-4-methyl-morpholinium Perchlorate (DET-MM(ClO_4_)) and 4-(4,6-diethoxy-1,3,5-triazin-2-yl)-4-methyl-morpholinium Tetrafluoroborate (DET-MM(BF_4_))

NMM (128 mg, 1.27 mmol) was added to a solution of CDET (232 mg, 1.14 mmol) and NaClO_4_ (155 mg, 1.27 mmol) in THF (4 mL) at 0 °C. After stirring for 1 h, the precipitate was collected, washed with THF, and dried under reduced pressure to give DET-MM(ClO_4_) as a white solid (180 mg, yield 43%). M.p. 181–185 °C; ^1^H NMR (300 MHz, D_2_O, 25 °C): δ = 4.55 (m, 6H), 4.08 ppm (d, 2H), 3.81 ppm (m, 4H), 3.47 ppm (s, 3H) 1.37 ppm (t, 6H); ^13^C NMR (75 MHz, D_2_O, 25 °C): δ = 173.18, 169.96, 67.10, 62.03, 59.96, 55.71, 13.24 ppm IR (KBr): ṽ = 1617, 1518, 1436, 1344, 1094, 823, 624 cm^−1^. (see Supplementary Materials for further details).

For DETMM(BF_4_) the same procedure was performed. DETMM(BF_4_) was obtained as a white solid (239 mg, yield 59%). ^1^H NMR (300 MHz, D_2_O, 25 °C): δ = 4.62 (m, 6H), 4.14 ppm (d, 2H), 3.87 ppm (m, 4H), 3.53 ppm (s, 3H), 1.42 ppm (t, 6H); ^13^C NMR (75 MHz, D_2_O, 25 °C): δ = 173.15, 169.89, 67.05, 61.99, 59.91, 53.24, 13.21 ppm IR (KBr): ṽ = 1613, 1517, 1424, 1341, 1084, 817, 611, 528 cm^−1^. (see Supplementary Material for further details).

#### 2.2.3. Cross-Linking of Bovine Collagen Powder with CDET/NMM

First, CDET (147 mg, 0.72 mmol) was dispersed in water (15 mL) and NMM (73 mg, 0.72 mmol) was added dropwise. After the complete dissolution of CDET in water, the solution was added to bovine collagen powder (300 mg, 0.36 mmol COOH_coll_), and dispersed in water (15 mL) under magnetic stirring. The stirring was continued for a further 4 h; then, the collagen powder was recovered by filtration, air dried, and analyzed by DSC.

#### 2.2.4. Tanning Tests

The pelts were received after standard beamhouse operations, and the pH of the cross-section was measured before experiments. The general procedure for the tanning tests was as follows: soaked delimed calf hides (50 wt/wt hide/water) were mixed in a drum together with a water solution (50 wt/wt hide/water) comprising 3.1 wt CDET and 1.9 wt NMM by the wt of the hide processed. After 4 h, the water was discharged, and the hide was washed with 100 %wt/wt water for 10 min. After tanning, a conventional post-tanning process was carried out using synthetic tannins and natural fat liquoring agents to produce crust upper leather. These leather samples were employed for physical–mechanical tests.

#### 2.2.5. Shrinking Temperature

The shrinking temperature (Ts) of CDET/*tert*-amine tanned leather was measured according to EN ISO 3380 (IULTCS/IUP 16, 2015). A leather sample was suspended vertically in water, and the rate of heating was maintained at 2 ± 0.2 °C/min. The Ts is the temperature at which the leather shrinks one-third of its original length.

#### 2.2.6. Physical–Mechanical Tests

The tensile strength and percentage elongation of tanned leather have been determined according to the standard IULTCS (International Union of Leather and Chemists Association) methods. Tensile strength and percentage extension were measured according to EN ISO 3376 (IULTCS/IUP 6, 2011), single edge tear load was determined according to EN ISO 3377-1 (IULTCS/IUP 40, 2011). Resistance to grain cracking was determined according to EN ISO 3378 (IULTCS/IUP 12, 2002). The mechanical properties of tanned leather were determined on eight parallel samples using a thermomechanical analyzer TMA (Instron series II Automated Materials Testing System). Prior to performing physical tests, all finished samples were conditioned according to standard EN ISO 2419 (IULTCS/IUP 1, 2012).

## 3. Results and Discussion

### 3.1. Amidation Reaction

**Scheme 1:**
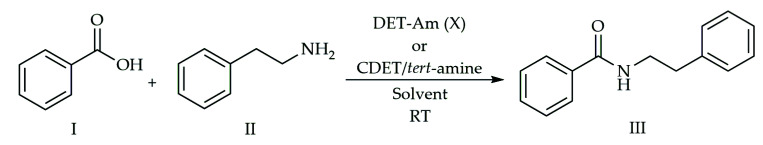
Dehydro-condensation reaction for the synthesis of amide *N*-phenethyl benzamide (III).

According to a consolidated protocol reported in the literature [31,46], the first experiments to verify the activity of CDET and a *tert*-amine were carried out using as standard reaction the amidation of benzoic acid (I) with 2-phenylethylamine (II) (Scheme 1).

Preliminary CDET/*tert*-amine activity was tested in different solvents. Relevant data are reported in Table 1 and referee to the average value of at least three replicates.

Methanol is the most employed solvent for condensation reactions in the presence of DMTMM or other triazine-based agents. With the intent to improve the environmental sustainability of the process, different nontoxic alcohols were used, such as ethanol and propan-2-ol, giving good even if slightly lower yields in amide III. Data reported in Table 1 show no significant difference between the alcoholic solvents used and the comparable data reported in literature with triazine condensation agents [46]. Further experiments were carried out in methanol, which has a lower boiling point and allows an easy recovery of the product.

Yields in amide III in water were lower (entry 4, Table 1), probably due to the low solubility of the reagents in this solvent and not to a decreased reactivity of the condensation agent. In fact, a reaction carried out in the same conditions as entry four by the addition of 10% vol of acetone to improve the solubility of the carboxylic acid, and the amine allowed us to recover the final amide in yields above 90% by 1 h (entry 5, Table 1).

Additionally, the influence of the *tert*-amines on the activity of CDET/*tert*-amine was tested (Table 2). *Tert*-amines employed in this work are reported in Figure 1.

When NMM was substituted with N-methylpiperidine (NMP), N-methylpyrrolidine (MPD), and trimethylamine (TMA), no significant difference in the activity of the condensation agent was detected since yields in III by 1 h are comparable (entries 1, 4, 7, 9 of Table 2). On the contrary, a significant decrease in III yields was observed with the CDET/NNDP (N,N’-Dimethylpiperazine) system (entry 11 of Table 2). 

According to literature data [44,46,47], counter anion exchange can favor the stability of triazine quaternary ammonium salts and thus improve their activity in condensation reactions. Raw et al. and Kitamura et al. report the synthesis and use of DMTMM(BF_4_) and DMTMM(ClO_4_) [47,48], while only one example is known of similar studies in the presence of in situ systems comparable to CDET/tert-amine [46]. Thus, further experiments were carried out to verify if counter anion exchange during condensation reaction could influence the reactivity of the CDET/tert-amine system.

Comparing yields in III achieved with CDET/NMM with CDET/NMM/NaClO_4_ or CDET/NMM/NaBF_4_, an overall decrease in yields in III was measured in both cases (compare entries 1, 2, and 3, Table 2). Thus, adversely to quaternary ammonium salts, the exchange of the chlorine atom with a perchlorate anion or tetrafluoroborate ion seems to decrease the reactivity of the in situ condensation agent. In fact, in agreement with this hypothesis, further experiments carried out in the presence of other *tert*-amines confirmed that the addition of NaClO_4_ or NaBF_4_ generally reduced yields in III, both after 15 min and 1 h. It may be supposed that the addition of ClO_4_^−^ or BF_4_^−^ leads to the formation of quaternary ammonium species such as DETMM(ClO_4_) or DETMM(BF_4_), which could be less reactive compared to CDET/*tert*-amine systems.

To verify this assumption, DETMM(BF_4_), DETMM(ClO_4_), and DETMM(Cl) were synthesized with a similar protocol reported in the literature for DMTMM [43,47,48]. Equivalent molar amounts of CDET, NMM, and NaClO_4_ or NaBF_4_ were mixed in THF for 1 h, and the white precipitate formed collected, allowing us to recover DETMM(ClO_4_) and DETMM(BF_4_) in, respectively 43% and 59% yield. On the contrary, DETMM(Cl) could not be isolated, and only decomposition by-products were recovered from the reaction mixture. DETMM(ClO_4_) and DETMM(BF_4_) were fully characterized by ^1^H, ^13^C NMR, FT-IR. The data are reported in the Supplementary Materials.

Then, DETMM(ClO_4_) and DETMM(BF_4_) were used as condensation agents for the synthesis of amide III (entries 13 and 14, Table 2), confirming that these condensation agents, if formed, have lower reactivity compared to CDET/*tert*-amine, probably because of their poor solubility. In the presence of CDET/NMM, yields in III by 1 h are around 90%, while in the presence of DETMM(ClO_4_) and DETMM(BF_4_) yields did not exceed 80% (compare entries 1, 13, and 14, Table 2). These data agree with previous studies on CDMT in situ systems and seem to confirm that a different reaction pathway occurs when in situ protocol is adopted compared to the use of preformed triazine quaternary ammonium salts. Further evidence of these different behaviors clearly emerges also from these studies since CDET/NMM proved to be an efficient condensation agent while DETMM could not even be synthesized. In other words, the use of 2-chloro-4,6-dialcoxy-1,3,5-triazine in the presence of a *tert*-amine does not lead to the formation of the corresponding quaternary ammonium salt, which then reacts, but to direct formation of the active ester between the carboxylic acid and the triazine. The presence of NaClO_4_ or NaBF_4_, on the other hand, favors the formation of the quaternary ammonium salt and, in this case, yields an amide decrease. This step is known to be the rate-determining step, and therefore, in situ systems tend to be more reactive, even when the corresponding quaternary ammonium salt cannot be synthesized. Further studies are in progress to substantiate this hypothesis.

Thus, according to the data reported in Table 1 and Table 2, CDET/*tert*-amine are active and easy to use systems that offer a considerable number of advantages not only compared to DET-Ams(X) but also with respect to many amidation protocols used today, such as carbodiimides, uranium, or guanidinium salt derivatives [49].

### 3.2. Cross-Linking Reaction of Collagen by CDET/NMM

Further experiments were carried out with CDET/*tert*-amine systems to verify their efficiency as collagen cross-linking agents. According to literature [31,46], bovine collagen powder (BCP) was used as standard reference substrate for preliminary cross-linking (or tanning) tests.

**Scheme 2:**
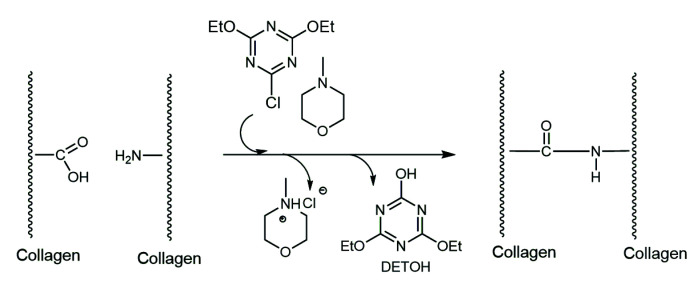
Cross-linking of collagen in the presence of CDET/*tert*-amine system.

Research shows that 1.0 g of BCP contains 1.2 mmol of free carboxylic acid groups (COOH_coll_) and 0.8 mmol of free amine groups (NH_2_coll) [50]. In the presence of a condensation agent such as CDET/*tert*-amine, these functional groups react, forming cross-linked amide bonds (Scheme 2) and consequently increase the thermal stability of the treated collagen. Generally, BCP has a Ts of about 50–55 °C, while after tanning Ts above 75–80 °C should be achieved for leather to be further processable [28,51].

BCP shrinking temperature (Ts) was measured by DSC analysis (see Supplementary Materials) before and after the reaction to evaluate the cross-linking efficiency of the CDET/*tert*-amine systems tested [52,53]. Cross-linking experiments were carried out according to the protocol optimized in our previous work [30,31] by adding different CDET/*tert*-amine systems to a water suspension of BCP; after 4 h collagen is filtered, washed, dried, and analyzed by DSC.

Preliminary tests were carried out with a molar ratio between COOH_coll_ and CDET of 1/2 (mol/mol). Results achieved with the best-performing CDET/*tert*-amine as condensation agents are reported in Table 3.

According to data reported in Table 3, CDET/NMM allowed us to achieve Ts values comparable to DMTMM (Ts of about 82–87 °C) [30]. From an environmental point of view, CDET has several advantages compared to DMTMM. First, DMTMM is obtained by the reaction of CDMT and NMM in THF. According to the protocol reported in this work, this synthetic step is not required with a significant reduction in solvent and energy consumption, processing time, and reduction in DMTMM yield. CDET and the *tert*-amine are introduced directly in the reaction mixture or tanning bath. Additionally, methanol is required for the synthesis of CDMT, while much safer ethanol is used to prepare CDET.

Interestingly, CDET/TMA gave Ts values even higher (Ts = 90 °C), one of the best results reported with a chrome-free tanning agent [15]. Nevertheless, the high volatility and strong smell of this amine make it rather impossible to use at an industrial scale. CDMT/MPD gave satisfactory results, reaching Ts of about 83 °C. However, this cross-linking system was not further tested due to the relevant risk phrases of MPD. Thus, only CDET/NMM was further employed for tanning tests on calf hides.

Preliminary tests were performed in small drums, using 25 × 20 cm^2^ size delimed calf hide samples (ca. 200 g) and then in larger drums loaded with one hide (about 10 kg) (Table 4).

Tanning tests were carried out under different conditions, and the shrinking temperature (Ts) of tanned hides was measured to evaluate CDET/NMM tanning activity according to a widely used protocol EN ISO 3380 (IULTCS/IUP 16, 2015) (for DSC analysis profile see Supplementary Materials).

In the literature, it is widely reported that DMTMM does not require buffers, achieving best results when pH falls in a range between 5.0 and 9.0 pH range [14]. This, in fact, is one of the key environmental benefits of DMTMM since chrome tanning requires a pickling phase (acidification) to be carried out just before the addition of chrome salts, during which the pH of the tanning bath is lowered to pH 2.5. This is required to allow chrome species to penetrate the collagen hide and react uniformly inside the whole section. Adversely, when hides are placed in such a strong acid environment, they tend to swallow to the point that deterioration may occur. Over 8 %wt of NaCl by weight of the hide processed is added to control this phenomenon. These high quantities of Cl^−^ contribute to the increased costs of wastewater management and the environmental impact of the process.

Accordingly, three different experiments were carried out at pH 5.0, 7.0, and 9.0 to verify if CDET/NMM could be used for tanning hides without pickling. Results obtained were comparable, giving Ts within 75–77 °C, confirming that both DMTMM and CDET/NMM could be used without any pH control, within a pH range of 5.0–9.0. On the contrary, no cross-linking activity was observed in experiments carried out at pH ≤ 3.0 or pH ≥ 10.0. According to these findings, all further tests were carried out at pH = 7 (Entries 1–4 Table 4).

For comparison reasons, the first experiments were carried out in the same reaction conditions employed with DMTMM. Thus, an overall amount of 5 %wt of tanning agent was used by weight of leather processed (CDET/NMM 3.1/1.9 wt/wt by wt of the hide).

Different experiments were then carried out with decreasing quantities of tanning agents, as reported in Table 4. When CDET/NMM was used as a tanning agent in 3.1/1.9 wt by wt of processed hide, Ts of 77 °C were measured after 4 h. If higher quantities of CDET/NMM were added, a gradual increase in Ts was observed in very short reaction times, reaching Ts of 82 °C in 2 h with 4.5/2.7 wt/wt of CDET/NMM by weight of treated hide (Entry 3 and 4 in Table 4). Experiments carried out with higher quantities of tanning agents gave no significant variation in Ts or in reduction of processing time.

According to us, the best results were achieved with CDET/NMM in 3.1/1.9 %wt/wt by weight of processed hide, in 18 h, reaching Ts of 80 °C, since the lowest amounts of tanning agents were employed even if with longer processing times and slightly lower Ts. In fact, from an industrial point of view, since the leather industry processes high quantities of product (125.000.00 m^2^ yearly only in Italy), a reduction of a few %wt of tanning agent used is relevant.

Physical–mechanical characterization of leather samples produced with CDET/NMM in 3.1/1.9 %wt/wt for wt of the processed hide are reported in Table 5.

The overall physical–mechanical properties, such as tensile and tear strength, elongation at break, and resistance to grain cracking obtained using CDET/NMM, are comparable with conventional chrome crust leather and comply with the standard requirements for upholstery leather [34,53,54].

## 4. Conclusions

In conclusion, the possibility to improve the sustainability of the leather industry should be one of the driving forces to restore leather as an alternative to synthetic materials, for example, in the automotive, textile, and apparel sectors. In this connection, in this work, different CDET/*tert*-amine systems were tested as alternative chrome-, formaldehyde-, and phenol-free tanning agents. The efficacy of CDET/*tert*-amine systems was first verified starting from benzoic acid and 2-phenylethylamine to produce the corresponding amide. Then, most active CDET/*tert*-amine systems were further tested as collagen cross-linking agents. An increase in collagen shrinking temperature was measured to evaluate the efficiency of the different triazine systems used. CDET/NMM and CDET/TMA gave very good Ts, respectively up to 85 °C and 90 °C, comparable to some of the most efficient tanning agents employed industrially today, such as aldehydes and natural or synthetic tannins. The best results were achieved with CDET/NMM in 3.1/1.9 wt/wt of the hide processed, in 18 h reaching Ts of 80 °C, and producing a crust leather complying with the standard requirements for upholstery leather. In conclusion, CDET/*tert*-amine system is a very active, versatile, simple, and environmentally sustainable alternative to many conventional condensation agents employed today, totally metal-, aldehydes-, and phenol-free.

## 5. Patents

Patent application number WO2016103185A3 was filed with the Italian Patent and Trademark Office (UIBM).

## Figures and Tables

**Table 1:** Condensation reaction of I and II in the presence of CDET/NMM in different solvents.

Entry	Solvent	Coupling Agent ^(a)^	Yield (%) ^(b)^ (15 min/60 min)
1	methanol	CDET/NMM	88/91
2	ethanol	CDET/NMM	86/89
3	propan-2-ol	CDET/NMM	79/86
4	H_2_O	CDET/NMM	42/50 ^(c)^
5	H_2_O/Acetone (90/10)	CDET/NMM	88/90

Reaction conditions: benzoic acid (I): 1.3 mmol, phenylethylamine (II): 1.2 mmol, Solvent: 6 mL, T: 25 °C. ^(a)^ Coupling agent: 1.3 mmol of 2-chloro-4,6-diethoxy-1,3,5-triazine and 1.3 mmol of NMM were added. ^(b)^ Yield in III was measured by GLC using mesitylene as internal standard (1.2 mmol). ^(c)^ Yields were measured on the weight of III recovered after workup.

**Table 2:** Condensation reaction of I and II in the presence of CDET/*tert*-amine with different counter anions.

Entry	Coupling Agent ^(a)^	Counter Anion	Yield (%) ^(b)^ (15 min/60 min)
1	CDET/NMM	Cl^−^	88/91
2	CDET/NMM	ClO_4_^−^	57/69
3	CDET/NMM	BF_4_^−^	76/78
4	CDET/NMP	Cl^−^	76/85
5	CDET/NMP	ClO_4_^−^	57/59
6	CDET/NMP	BF_4_^−^	62/66
7	CDET/MPD	Cl^−^	79/90
8	CDET/MPD	ClO_4_^−^	66/68
9	CDET/TMA	Cl^−^	83/84
10	CDET/TMA	ClO_4_^−^	72/87
11	CDET/NNDP	Cl^−^	64/69
12	CDET/NNDP	ClO_4_^−^	51/70
13	DETMM	ClO_4_^−^	71/80
14	DETMM	BF_4_^−^	71/73

Reaction conditions: benzoic acid (I): 1.3 mmol, phenylethylamine (II): 1.2 mmol, Solvent: 6 mL methanol, T: 25 °C. ^(a)^ Coupling agent: 1.3 mmol of CDET and 1.3 mmol of *tert*-amine were added. ^(b)^ Yield in III was measured by GLC using mesitylene as internal standard (1.2 mmol).

**Table 3:** Shrinkage temperature of collagen cross-linking by different CDET/*tert*-Amine systems.

Entry	Coupling Agent ^(a)^	Ts (°C) ^(a)^
1	CDET/NMM	85
2	CDET/MPD	83
3	CDET/TMA	90

Reaction conditions: 300 mg of bovine collagen powder (0.36 mmol COOH_coll_), 147 mg of CDET (0.72 mmol), *tert*-amine (0.72 mmol), 15 mL of water, T: 25 °C, t: 4 h. ^(a)^ Ts was measured by DSC analysis (see also Supplementary Materials).

**Table 4:** Tanning tests on calf hide in the presence of CDET/NMM system.

Entry	Hide (kg)	CDET/NMM ^(a)^	Water (kg)	t (h)	Ts (°C) ^(b)^
1	7.5	3.1/1.9	7.5	4.0	77
2	13	3.1/1.9	10	18.0	80
3	10	4.5/2.7	10	2.0	82
4	12	4.0/2.4	12	2.5	80

^(a)^ CDET/NMM: wt quantities used by wt of calf hide. ^(b)^ Ts was measured according to EN ISO 3380.

**Table 5:** Physical-mechanical properties of CDET/NMM and chrome tanned leather.

Test	CDET/NMM ^(a)^	Chrome ^(b)^
Tensile strength (N/mm^2^) ^(c,d)^	20.0 ± 0.5	25.0 ± 0.8
Tear strength (N/mm) ^(c,e)^	36.5 ± 0.3	40.0 ± 0.5
Elongation (%) ^(c,d)^	44.5 ± 1.0	46.0 ± 0.8
Resistance to grain cracking (N/mm^2^) ^(c,f)^	9.3 ± 0.2	10.0 ± 0.1

^(a)^ Crust leather samples prepared by tanning with 3.1/1.9 wt/wt CDET/NMM by weight of the hide processed and retanning with synthetic tannins (see experimental section). ^(b)^ Crust leather samples prepared by tanning with 5 %wt basic chromium sulfate and retanning with synthetic tannins. ^(c)^ Average of mean and cross backbone samples. ^(d)^ Measured according to EN ISO 3376 (IULTCS/IUP 6, 2011). ^(e)^ Measured according to EN ISO 3377-1 (IULTCS/IUP 40, 2011). ^(f)^ Measured according to EN ISO 3378 (IULTCS/IUP 12, 2002).

## Data Availability

Not applicable.

## Supplementary Materials

The following are available online at https://www.mdpi.com/article/10.3390/ma14113069/s1, Figure S1. ^1^H NMR spectrum of DET-MM (ClO_4_) in D_2_O, Figure S2. ^13^C NMR spectrum of DET-MM (ClO_4_) in D_2_O, Figure S3. FT-IR spectrum of DET-MM (ClO4) in KBr, Figure S4. ^1^H NMR spectrum of DET-MM (BF_4_) in D_2_O, Figure S5. ^13^C NMR spectrum of DET-MM (BF_4_) in D_2_O, Figure S6. FT-IR spectrum of DET-MM (BF_4_) in KBr, Figure S7. DSC spectra of collagen cross-linking by a. CDET/NMM system and b. CDET/MPD system.
