# Supplementary material for: Efficient Triazine Derivatives for Collagenous Materials Stabilization
Source: Materials (Basel). 2021 Jun 4;14(11):3069. doi: 10.3390/ma14113069 (PMC8200008; doi:10.3390/ma14113069)
Supplement: Supplementary file 1 [file materials-14-03069-s001.zip › materials-1210337-supplementary.pdf]

materials

Supplementary Materials

Efficient triazine derivatives for collagenous materials stabilization

Vanessa Gatto [1,2], Silvia Conca [1], Noemi Bardella [1], Valentina Beghetto [1,2*]

[1] Department of Molecular Sciences and Nanosystems, University Ca' Foscari of Venice, Via Torino 155, 30172 Mestre (VE), ITALY; vanessa.gatto@unive.it, silvia.conca@unive.it, noemi.bardella@unive.it, beghetto@unive.it
[2] Crossing S.r.l., Viale della Repubblica 193/b, 31100 Treviso (TV), ITALY; vanessa.gatto@crossing-srl.com, valentina.beghetto@crossing-srl.com
* Correspondence: beghetto@unive.it

Received: date; Accepted: date; Published: date

Table of Contents

I. DET-MM (ClO_4) characterization (pages 2-3)
Figure S1. ^{1}H NMR spectrum of DETMM(ClO_4).
Figure S2. ^{13}C NMR spectrum of DETMM(ClO_4).
Figure S3. FT-IR spectrum of DETMM(ClO_4).
II. DET-MM (BF_4) characterization (pages 4-5)
Figure S4. ^{1}H NMR spectrum of DETMM(BF_4).
Figure S5. ^{13}C NMR spectrum of DETMM(BF_4).
Figure S6. FT-IR spectrum of DETMM(BF_4).
III. DSC analysis of calf collagen and leather samples (pages 6-7)

I. DETMM(ClO_4) characterization

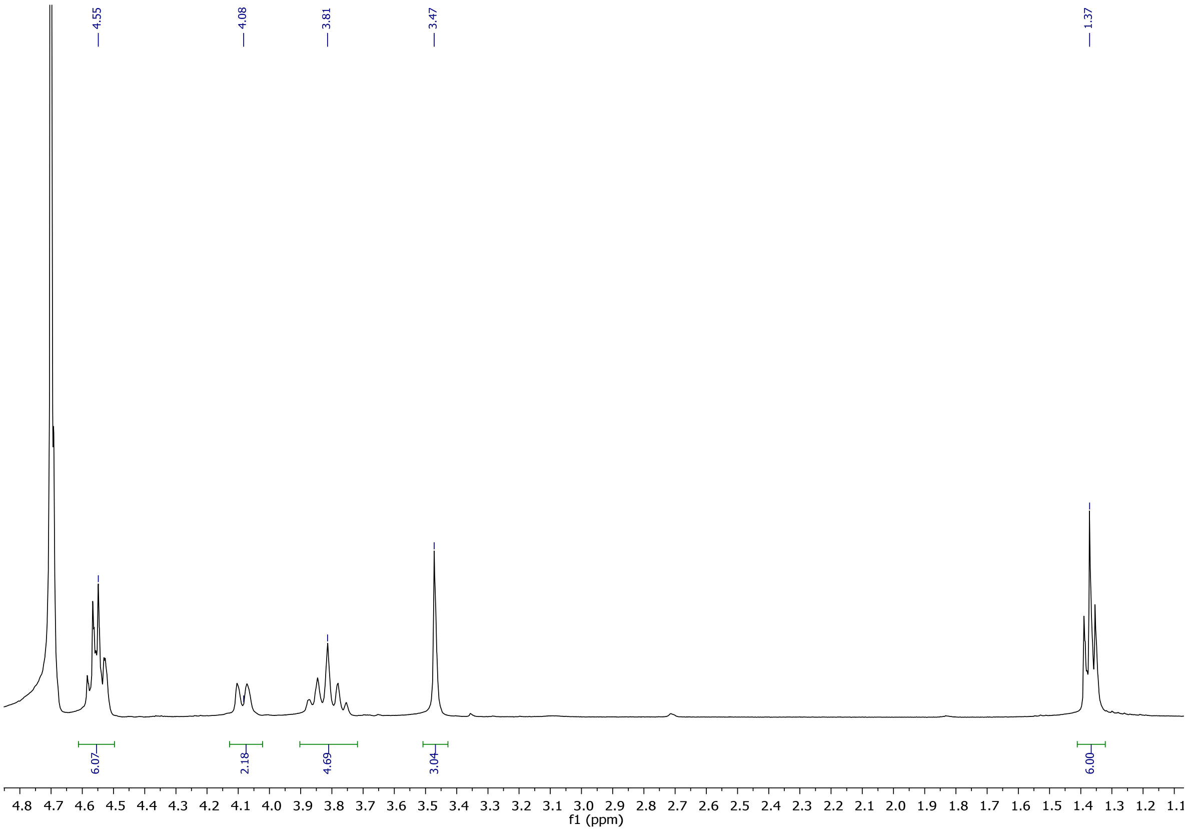

Figure S1. ^{1}H NMR spectrum of DETMM(ClO_4) in D_2O.

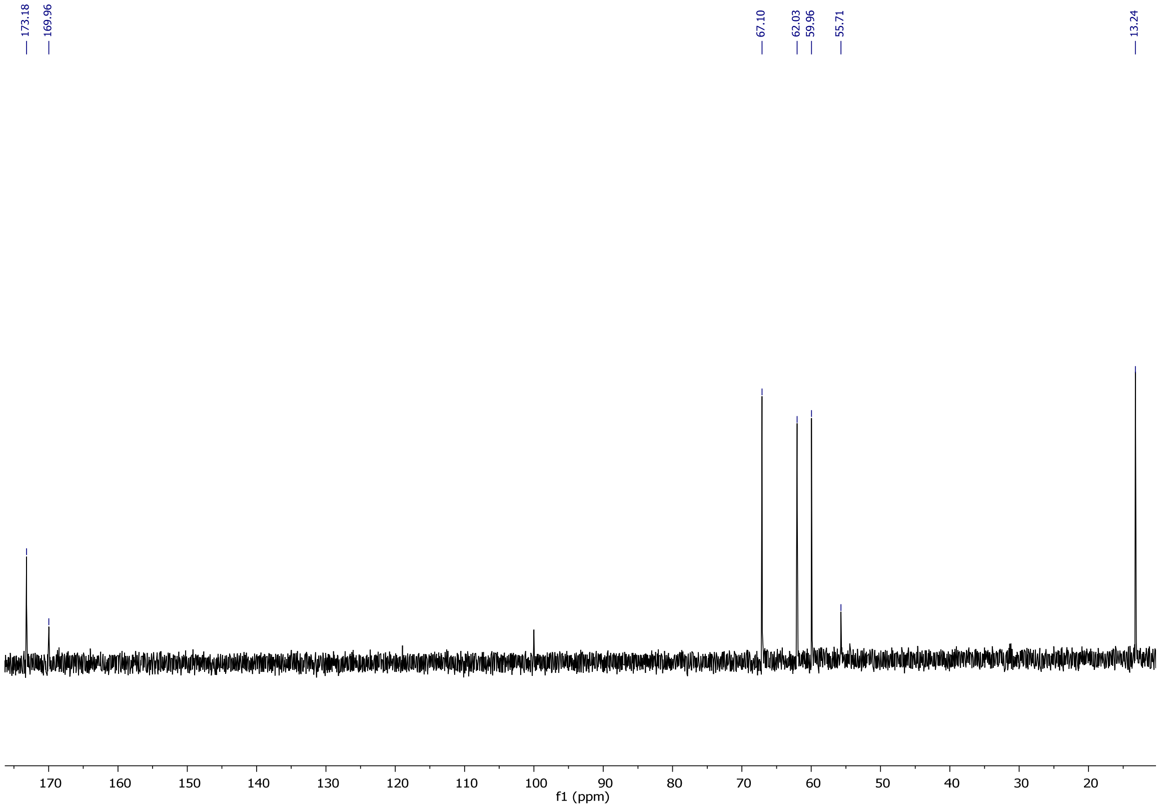

Figure S2. ^{13}C NMR spectrum of DETMM(ClO_4) in D_2O.

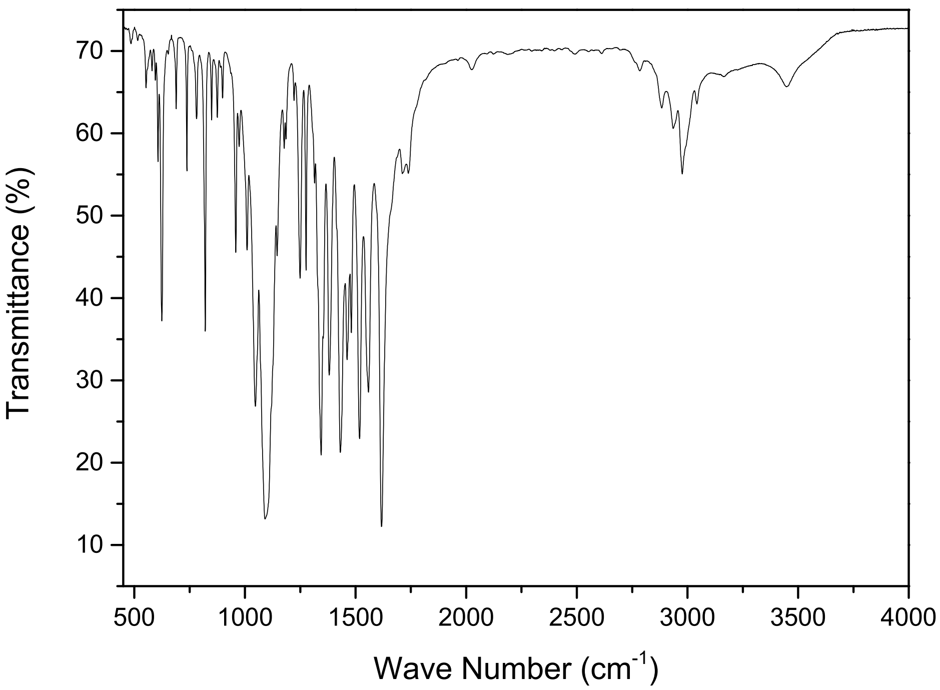

Figure S3. FT-IR spectrum of DETMM(ClO_4) in KBr.

II. DETMM(BF_4) characterization

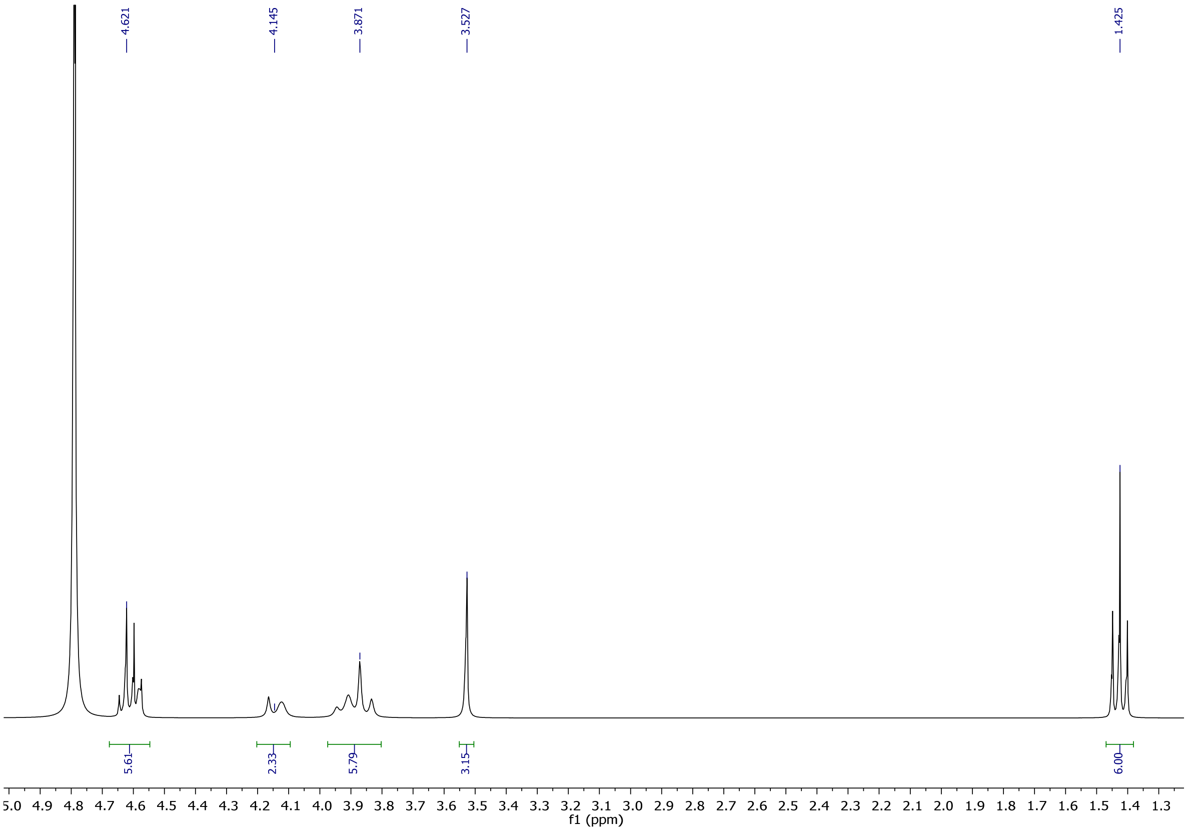

Figure S4. ^{1}H NMR spectrum of DETMM(BF_4) in D_2O.

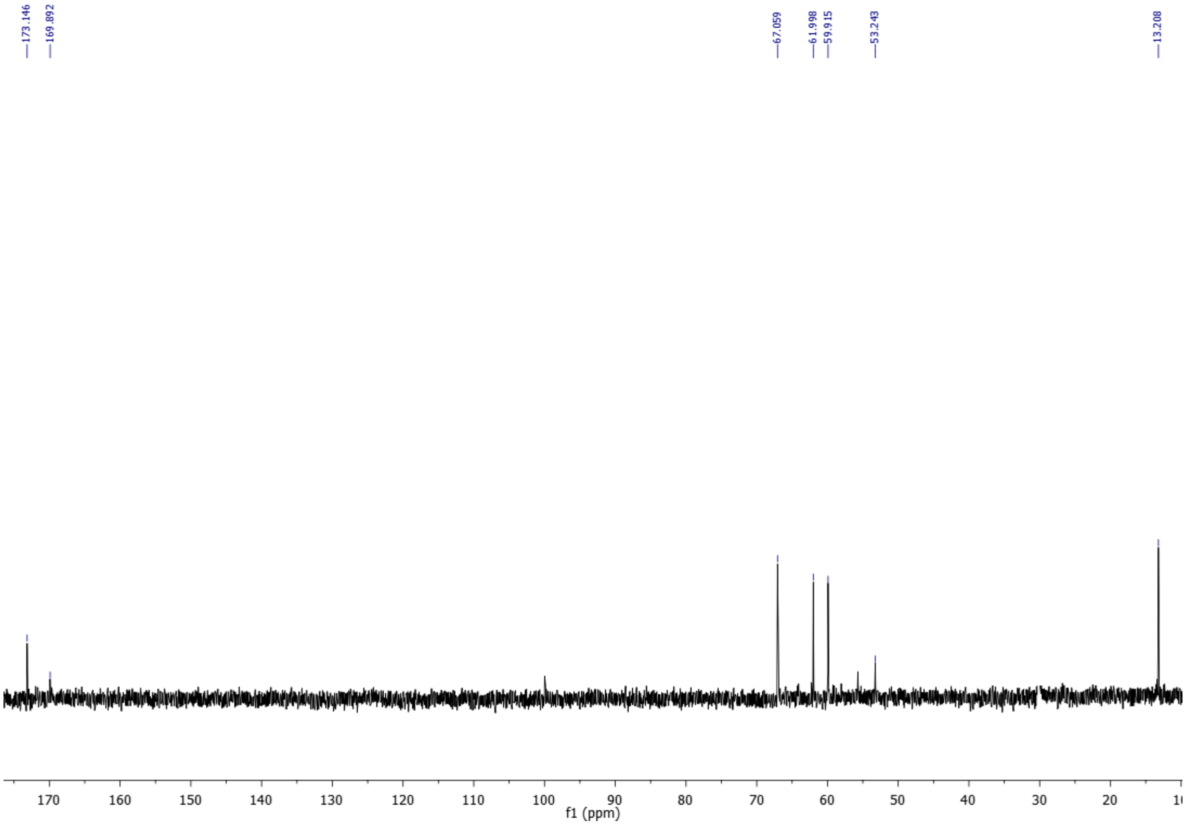

Figure S5. ^{13}C NMR spectrum of DETMM(BF_4) in D_2O.

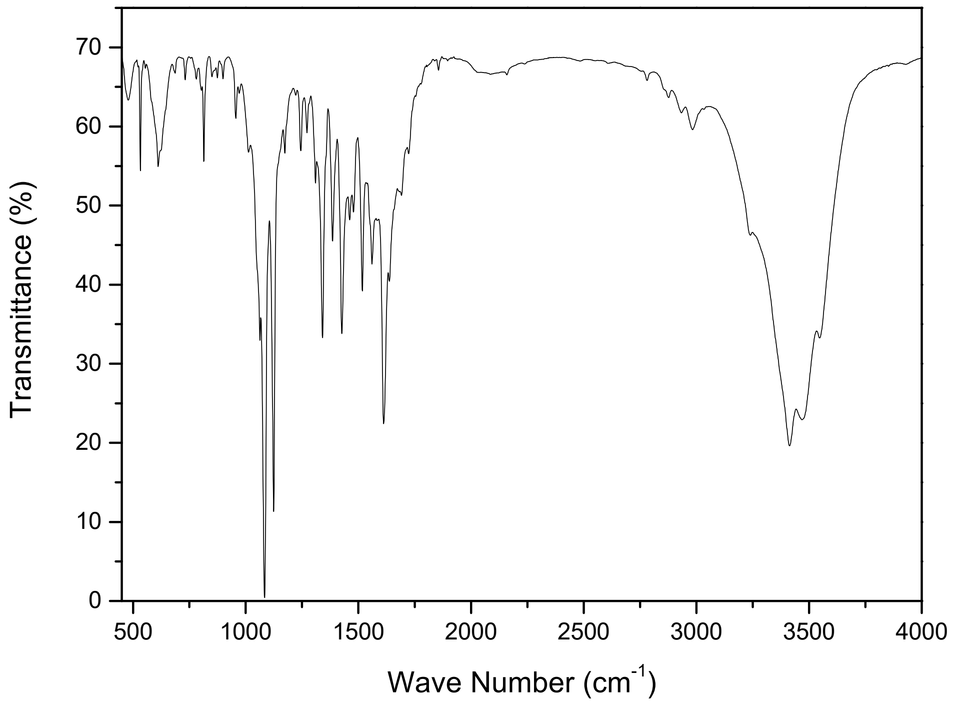

Figure S6. FT-IR spectrum of DETMM(BF_4) in KBr.

III. DSC analysis of calf collagen and leather samples

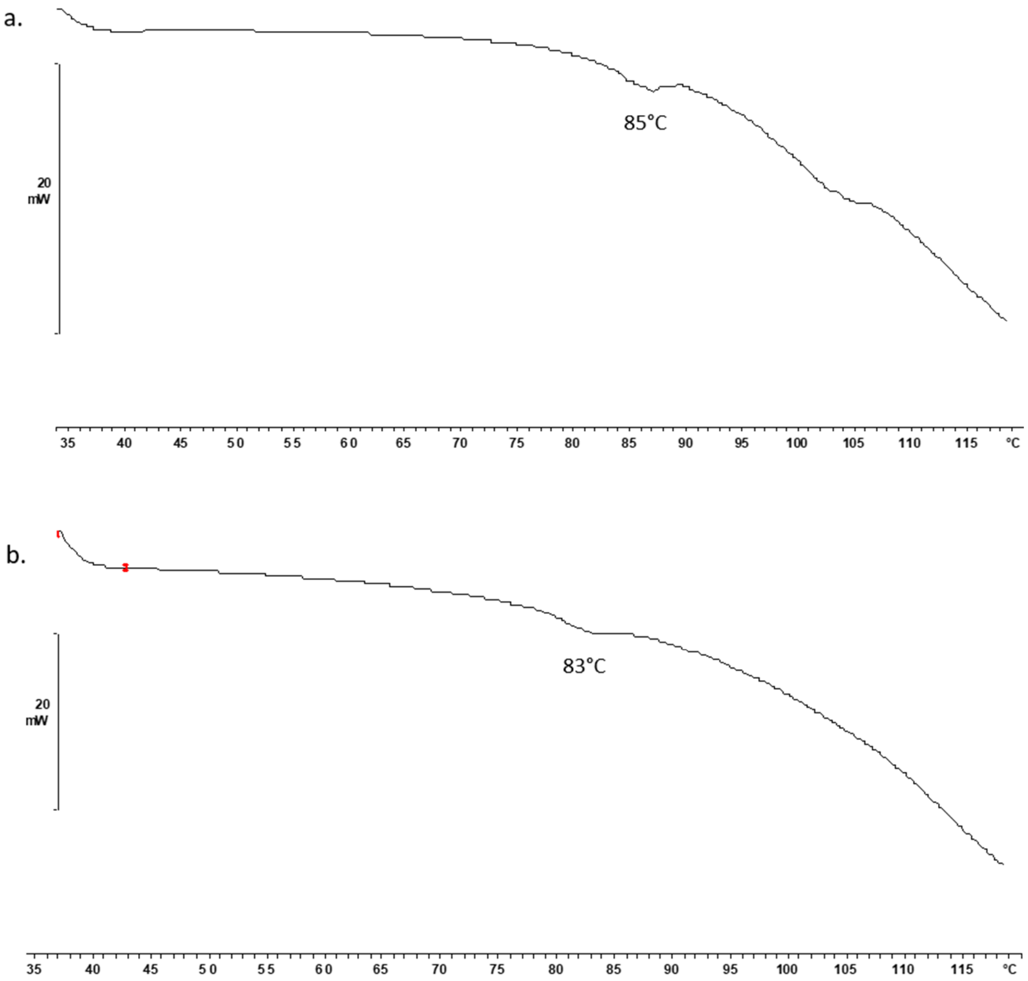

Figure S7. DSC spectra of collagen cross-linking by a. CDET/NMM system and b. CDET/MPD system.
